# Eosinophilic cytokines and small airway dysfunction in women with COPD: correlation with exposure to biomass smoke versus tobacco smoking

**DOI:** 10.5588/ijtldopen.24.0666

**Published:** 2025-09-10

**Authors:** R. Robles-Hernández, R. Pérez-Padilla, A. Ramírez-Venegas, Y. Velasco-Torres, M. Montaño, C. Ramos, A.S. Ramírez-GarciaLuna, O. Pérez-Bautista

**Affiliations:** ^1^ Departmento de Investigación en Tabaquismo y EPOC, Instituto Nacional de Enfermedades respiratorias ‘Ismael Cosío Villegas,’ Tlalpan Mexico City, Mexico;; ^2^ Laboratorio de Biología Celular, Departamento de Investigación en Fibrosis Pulmonar, Instituto Nacional de Enfermedades respiratorias ‘Ismael Cosío Villegas,’ Tlalpan Mexico City, Mexico;; ^3^ Servicio de Neumología, Hospital Universitario de Puebla BUAP, Puebla, Mexico;; ^4^ Hospital Medica Sur, Tlalpan, Mexico City, Mexico.

**Keywords:** airways obstruction, Th2 inflammation, small airway disease, biomass smoke, eosinophils, impulse oscillometry, tobacco smoking

## Abstract

**BACKGROUND:**

Chronic obstructive pulmonary disease (COPD) is a chronic inflammatory disease characterised by progressive airflow obstruction. Tobacco smoking is the leading cause of COPD (COPD-TS); however, chronic exposure to biomass smoke (COPD-BS) is the second risk factor. COPD-TS and COPD-BS are now considered different phenotypes, with COPD-BS having small airway dysfunction (SAD).

**OBJECTIVES:**

Our primary aim was to investigate the correlation between SAD and the serum levels of interleukins implicated in the maturation and recruitment of eosinophils in women with COPD-BS and COPD-TS.

**METHODS:**

This cross-sectional study included 25 women with COPD-BS and 25 women with COPD-TS, and the relationship with SAD was assessed by impulse oscillometry (IOS), alongside measurements of interleukins (IL-1ra, IL-2, IL-4, IL-8, IL-9, IL-13, and IL-17) and eotaxin using the multiplex test (Bio-Plex), in addition to eosinophils total blood.

**RESULTS:**

Blood eosinophil count, cytokines, and eotaxin were higher in COPD-BS relative to COPD-TS. IOS measurements were higher in COPD-BS than in COPD-TS. R5–R20 positively correlated with the concentrations of all measured cytokines noted if COPD groups were analysed separately (*P* < 0.01).

**CONCLUSION:**

Eosinophil counts and cytokines in COPD-BS positively correlate with small airway resistance as measured by IOS in R5–R20.

Chronic obstructive pulmonary disease (COPD) is a systemic inflammatory disease that causes progressive airflow obstruction in the lungs. Tobacco smoking (TS) is the leading cause of COPD (COPD-TS), but chronic exposure to biomass smoke (COPD-BS), mainly wood smoke, is a leading factor in women from low-income countries and vulnerable socio-economic populations.^1^ Several differences between COPD-BS and COPD-TS, including clinical and functional features, have been described as becoming distinct phenotypes for some experts.^2,3^ COPD-BS patients tend to have more small airway impairment than COPD-TS patients, demonstrated by both histological and functional points of view; nevertheless, we still have a limited understanding of the mechanisms involved in the damage to small airways.^4,5^ Airway inflammation in COPD-TS is a complex process. While inflammation is usually neutrophilic,^6^ up to one third of patients may present with eosinophilic inflammation, which may vary over time.^7^ There is some evidence showing the presence of eosinophilic inflammation in COPD-BS.^8–10^ Therefore, it is plausible that the differences in both types of COPD can be explained, at least partially, by the differences in the local and systemic inflammatory profile and the polarisation towards a Th2 response. Some COPD exacerbations show a partial predominance of eosinophils. However, their pathophysiologic role in COPD is not fully understood. Some studies analysed the inflammatory profile of COPD-BS and COPD-TS and found that patients with COPD-BS had higher eosinophilic profile or Th2 markers than patients with COPD-TS.^9–11^ However, Golpe et al.^12^ did not find this predominant Th2-type inflammation in COPD-BS. Therefore, evidence supporting a predominance of eosinophilic inflammation in patients with COPD-BS is limited and heterogeneous.

We hypothesise that women with COPD-BS have increased airway resistance associated with eosinophilic inflammation and higher levels of cytokines responsible for these effects compared with women with COPD-TS. Consequently, this study aimed to evaluate small airway disease by impulse oscillometry (IOS) and its correlation with serum levels of interleukins involved in eosinophil maturation and recruitment, comparing patients with COPD secondary to smoking and exposure to BS.

## METHODS

### Study population

This study was done at Instituto Nacional de Enfermedades Respiratorias Ismael Cosío Villegas (INER), a referral centre for respiratory diseases in Mexico City for uninsured individuals. Participants were recruited at the COPD clinic from a regularly followed cohort.^13^

For the inclusion of patients, the previous COPD diagnosis was considered. The diagnosis of COPD was defined according to European Respiratory Society/American Thoracic Society (ERS-ATS) standards, which include chronic symptoms, significant exposure, and a post-bronchodilator forced expiratory volume in the first second/forced vital capacity (FEV1/FVC) ratio <0.7 on spirometry^14^; COPD was significant when exposure histories reported the history of TS > 10 pack-years, and cumulative exposure to BS was expressed as >100 hours-year.^15^ Only women participants we recruited for a better comparison because they tend to be more exposed to BS than men.^16^ We excluded treatment with inhaled corticosteroids and paired by the severity of GOLD FEV1 classification. Patients who had no self-reported active exposure to biomass smoke or tobacco smoke in the last year so that active exposure would not be a confounding factor in the measurement of cytokines. Patients with a history of other conditions were excluded, such as asthma, allergic rhinitis, atopic dermatitis, TB bronchiectasis, or any other non-pulmonary disease. All participants were clinically stable and without exacerbation for at least 6 weeks before the study. The study’s power was estimated to find a difference in the number of eosinophils at least 20%, in concordance with Salvi et al.’s results.^17^

Demographic, anthropometric, and clinical data were collected, and the average hours per day of exposure while cooking were multiplied by the number of years of exposure by clinical interview. We also applied a standardised Spanish version of the ATS questionnaire.^18^

### Pulmonary function tests

Pre- and post-bronchodilation spirometry was performed in all women following the procedures recommended by the ATS/ERS, with a dry rolling seal volume spirometer (Sensormedics, Yorba Linda, CA, USA). We analysed the FEV1 and FVC expressed as percentage of predicted according to Mexican standard reference equations.^19^

### Impulse oscillometry

IOS was performed before spirometry using the digital system MS-IOS (Jaeger, Würzburg, Germany), following the ERS protocols and recommendations.^20^ Subjects sat comfortably upright and were asked to wear a nose clip and apply manual compression to the face to minimise cheek vibration and air leakage, coupled with an antibacterial filter. Resistance at 5 Hz (R5), 20 Hz, resonant frequency (Fres), and area under the reactance curve between Fres and 5 Hz (AX) were obtained. Some surrogated indicators of small airway function were the difference between resistance at 5 and 20 Hz (R5–R20) and the AX. Acceptability criteria for the recordings included the lack of visually detected artefacts and coherence of at least 0.6 at 5 Hz and 0.9 at 10 Hz.

### Blood samples

Eosinophils were counted by routine complete blood count, expressing their number of cells per Litre. Serum was obtained from whole blood in all women; for each sample, 5 mL of whole blood was collected in tubes free of anticoagulants (BD Vacutainer, Becton, Franklin Lakes, NJ, USA). Samples were collected in the morning with at least 8 h of fasting. Samples were incubated in a vertical position for 1 h at room temperature and then centrifuged at 5,000 × g for 15 min to obtain the serum, kept at −20°C until analysis.

### Measurement of serum cytokines

Concentration levels of 16 cytokines in the serum of the 50 women with COPD were analysed using the Bio-Plex Pro Human Cytokine Th1/Th2 Assay (171-AC500M; BioRad, Inc., Hercules, CA, USA). Eight cytokines (G-CSF, PECAM-1, VCAM-1, IL5, IL10, IL12, RANTES, and TNF-α) are involved in the blood’s eosinophil maturation, differentiation, and survival. The remaining cytokines were selected to evaluate maturation (IL5 and G-CSF), differentiation, and survival in the blood (PECAM-1 and VCAM-1). The Regulated measured the migration of eosinophils to the lungs on Activation, Normal T cell Expression and Secreted (RANTES) and CCL11. The pro-inflammatory response generated in the lungs and derived by eosinophils was quantified by cytokines (IL-1Rα1, IL2, IL4, IL5, IL8, IL9 IL10, IL12, IL13, IL17), chemokines (RANTES and CCL11), and growth factors (TNF-α). Cytokine concentration was expressed as picograms per millilitre of serum.

### Statistical analysis

Comparison between COPD groups was assessed using Student’s *t*-test or by a non-parametric test. The serum cytokine distribution was non-normal and described using median and interquartile ranges. Spearman’s correlation coefficient was performed to analyse the relationship between serum concentration of inflammatory cytokines and R5–R20. A *P* value < 0.05 was considered statistically significant. All analyses were performed using the GraphPad Prism (v. 6.1, GraphPad Software, Inc., San Diego, CA, USA).

### Ethical statement

This protocol is derived from project C49-17, approved by the local ethics committee.

## RESULTS

### Characteristics of women in the study

Table 1 shows clinical data for women with COPD. The average exposure to BS was 366 ± 30 hour-years, while in TS, the mean cumulative tobacco consumption was 26.4 ± 8 pack-years, average years without active exposure to tobacco or wood smoke were 9.5 and 10.2, respectively, with no significant differences (*P* = 0.42). We found no differences in FEV1 between groups and neither response to bronchodilators in FEV1 or FVC. The eosinophil count was higher in COPD-BS compared with COPD-TS; the measurements in lymphocyte, leucocyte, and neutrophil counts found no differences between the groups (Table 1).

**Table 1.** Anthropometric, clinical, and physiological characteristics of women in the study.

Parameter	COPD-TS (n = 25)	COPD-BS (n = 25)
Age (years)^A^	69 ± 7	73 ± 6*
Height (cm)^A^	153.9 ± 8	147.6 ± 8*
Smoking history (pack-years)^A^	26.4 ± 8	—
Cumulative exposure to biomass smoke (hour-years)^A^	—	366 ± 3
Year to exposition BS or TS	9.5 ± 3.4	10.2 ± 5.6
BMI (Kg/m^2^)	26.7 ± 5	29.2 ± 3*
GOLD 1^B^	5 (20)	5 (20)
GOLD 2^B^	12 (48)	13 (52)
GOLD 3^B^	8 (32)	7 (28)
FEV_1_/FVC ratio^A^	58.1 ± 3	59.1 ± 4
FEV_1_%^A^	68.8 ± 5	69.9 ± 4
FEV_1_ L^A^	1.06 ± 0.52	1.07 ± 0.34
FVC%^A^	79 ± 16	73 ± 25
FVCL^A^	1.93 ± 0.44	1.73 ± 0.5
FVC response (ml)^A^	89 ± 210	90 ± 210
FEV1 response (ml)	80 ± 90	70 ± 160
Eosinophil count (cells/µL)^A^	220 ± 100	300 ± 80**
Leucocytes (cells/µL)^A^	7,000 ± 1,800	6,700 ± 1,900
Neutrophils (cells/µL)^A^	4,800 ± 2,600	4,400 ± 1,700
Lymphocytes (cells/µL)^A^	1,900 ± 1,200	1,900 ± 550

Pack-year was obtained by multiplying the number of packs of 20 cigarettes smoked per day by the number of years the person has smoked; hour-years was calculated by multiplying the number of years cooking with wood stoves by the average daily hours spent in the kitchen. Statistical analysis was performed using Mann–Whitney *U* test for continuous variables and Fisher’s test for categorical variables.

Significance: *P < 0.05. **P < 0.01.

BMI = body mass index; COPD-BS = chronic obstructive pulmonary disease by exposure to biomass smoke; COPD-TS = COPD by tobacco smoking; FEV1%P = forced expiratory volume in the first second (% predicted); FVC = forced vital capacity; FEV1/FVC ratio = forced expiratory volume in the first second (% predicted)/forced vital capacity ratio.

A Data are expressed as median and ± interquartile range.

B Data are expressed as number and % in parentheses.

### Serum cytokine levels in women with COPD

**Figure 1.**
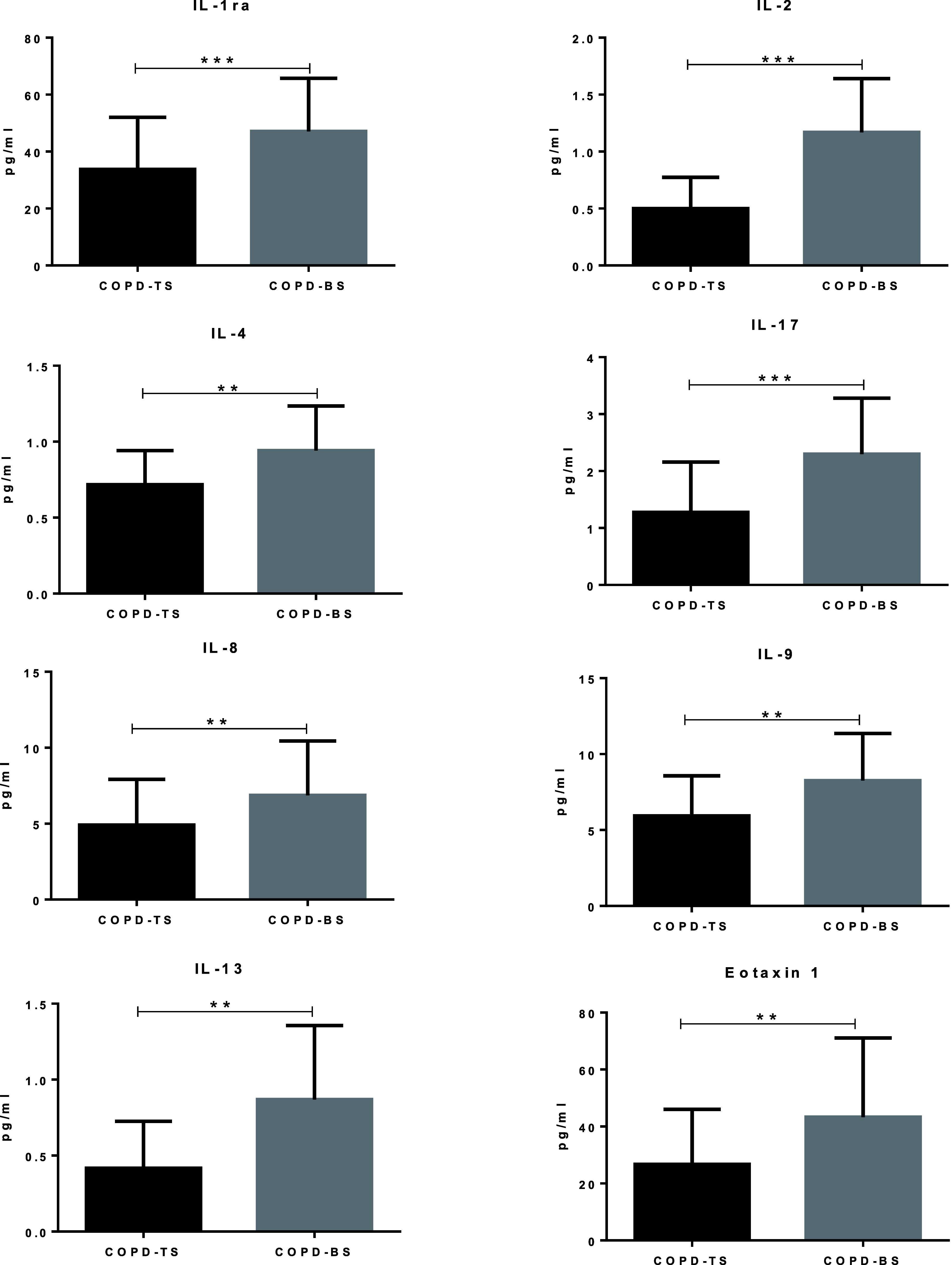
Serum concentration levels of cytokines in COPD-TS and COPD-BS women. Individual data are expressed as points, indicating the median (interquartile range). Statistical analysis was performed using the Kolmogorov–Smirnov test. COPD-BS = chronic obstructive pulmonary disease by exposure to biomass smoke; COPD-TS = COPD by tobacco smoking; IL = interleukin; IL-1ra = interleukin-1 receptor antagonist, IL-1RA/IL-1Rα/IL-1Rα1. *P < 0.5, **P < 0.002, ***P < 0.0001.

Eight cytokines (G-CSF, PECAM-1, VCAM-1, IL5, IL10, IL12, RANTES, and TNF-α) had more than 50% of the values below the detection limit and were not further analysed. Statistical analyses indicated that concentration levels of the remaining eight cytokines under study showed significant differences between study groups, with higher levels of IL-1Rα1, IL2, IL4, IL8, IL9, IL13, IL17, and CCL11 for COPD-BS than COPD-TS (Table 2; Figure 1).

**Table 2.** Comparative data of the serum concentration of cytokines in the serum of women with COPD.

Cytokine, pg/mL	COPD-TS (n = 25)	COPD-BS (n = 25)
IL-1ra	24.76 (23.11–36.14)	40.39 (30.39–59.76)***
IL-2	0.51 (0.31–0.55)	1.31 (0.76–1.54)**
IL-4	0.68 (0.60–0.77)	0.91 (0.83–1.17)**
IL-8	4.28 (2.82–4.93)	6.11 (4.80–7.69)**
IL-9	5.38 (4.25–6.96)	7.54 (6.14–9.63)**
IL-13	0.34 (0.20–0.68)	0.82 (0.68–0.94)**
IL-17	0.88 (0.56–1.71)	2.21 (1.71–2.88)***
Eotaxin	20.04 (15.48–29.47)	38.15 (25.69–49.26)**

Data are expressed as median and interquartile range. Hypothesis testing was conducted using Kolmogorov–Smirnov’s test.

**P < 0.002, ***P < 0.0001.

COPD-BS = chronic obstructive pulmonary disease by exposure to biomass smoke; COPD-TS = COPD by tobacco smoking; IL = interleukin; IL-1ra = interleukin-1 receptor antagonist, IL-1RA/IL-1Rα/IL-1Rα1.

### IOS analysis

R5, AX, and R5–R20, indicators of small airway resistance, were higher in the COPD-BS than in the COPD-TS group; X5 was significantly lower in COPD-TS (*P* < 0.05), as shown in Table 3. There was no significant difference in R20 among study groups.

**Table 3.** IOS parameters between COPD groups.

IOS Parameter	COPD-TS (n = 25)	COPD-BS (n = 25)
R5 (kPa·s·L^−1^)	0.66 ± 0.33	0.83 ± 0.46*
R5%p (kPa·s·L^−1^)	185 ± 56	268 ± 90*
R20 (kPa·s·L^−1^)	0.37 ± 0.10	0.38 ± 0.13
R20%p (kPa·s·L^−1^)	12.21 ± 35	13.54 ± 30
R5–R20 (kPa·s·L^−1^)	0.29 ± 0.25	0.48 ± 0.26*
X5 (kPa·s·L^−1^)	−0.37 ± 0.32	−0.78 ± 0.23*
X5%p (kPa·s·L^−1^)	433 ± 190	640 ± 265.73*
AX (kPa·L^−1^)	3.8 ± 2.5	8.5 ± 3.5*

Data are expressed as means ± SD. The statistical analysis was probed normality and carried out using Student’s *t*-test.

*P < 0.05.

AX = reactance area; COPD-BS = chronic obstructive pulmonary disease by exposure to biomass smoke; COPD-TS = COPD by tobacco smoking; IOS= impulse oscillometry; R5 = resistance at 5 Hz [total]; R20 = resistance at 20 Hz [central]; R5–R20 = resistance at 5 Hz–resistance at 20 Hz [peripheral]; X5 = reactance at 5 Hz; %p = percent of the predicted.

### Correlation between IOS and inflammatory cytokines

**Figure 2.**
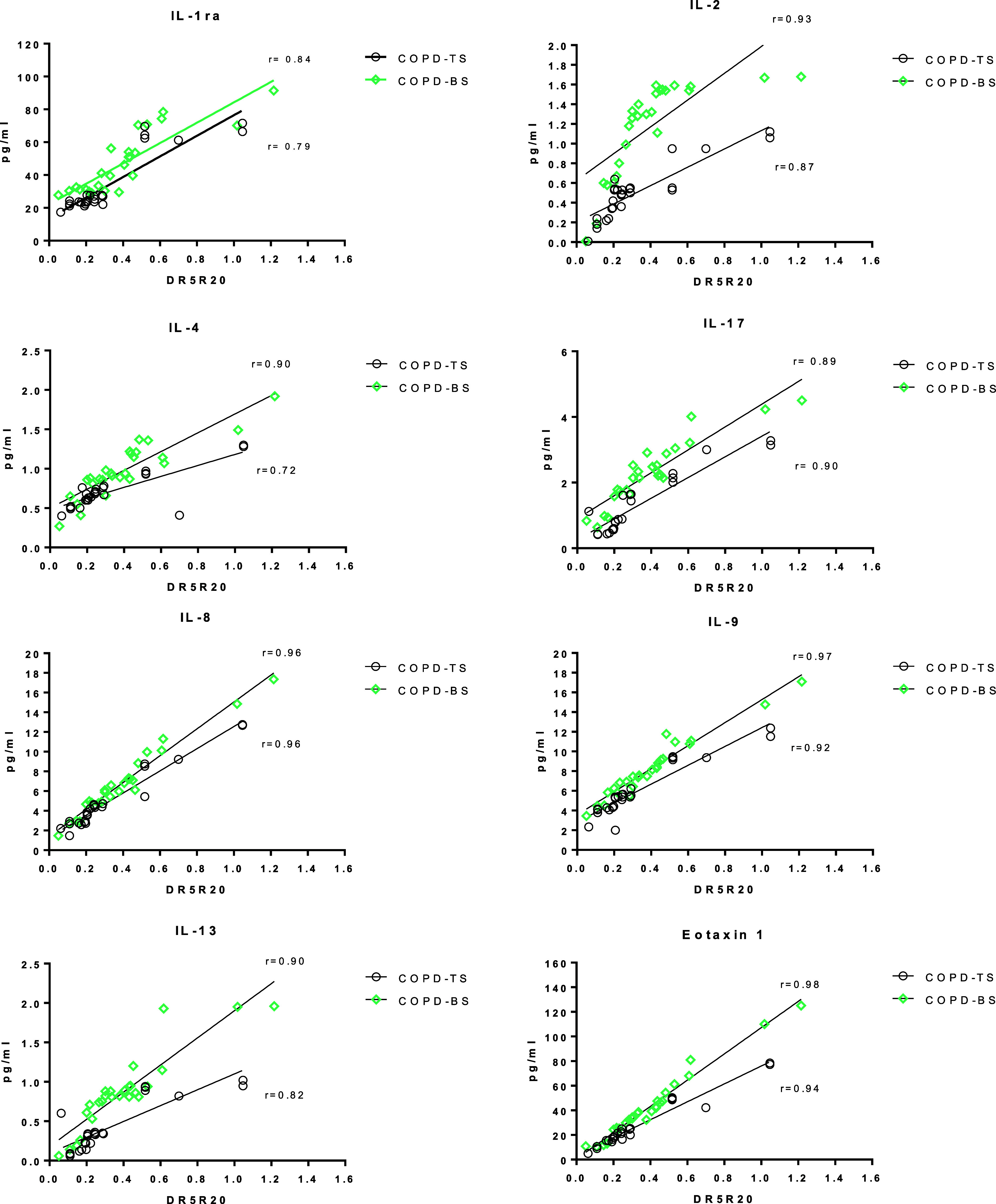
Correlation analysis between serum concentration of IL-1ra, IL-2, IL-4, IL-8, IL-9, IL-13, IL-17, and eotaxin with R5–R20 (IOS). COPD groups were analysed independently. Individual data are expressed as points. COPD-BS = chronic obstructive pulmonary disease by exposure to biomass smoke; COPD-TS = COPD by tobacco smoking; IL = interleukin; IL-1ra = interleukin-1 receptor antagonist, IL-1RA/IL-1Rα/IL-1Rα1; R5 = the resistance of the respiratory system at 5 Hz; R20 = the resistance of the respiratory system at 20 Hz. *P* < 0.01 in all cases.

Figure 2 presents the correlations between serum cytokines and R5–R20. We found a positive correlation between IL-1ra, IL-2, IL-4, IL-8, IL-9, IL-13, IL-17, and eotaxin and R5–R20 in both COPD groups, analysed separately. *P* < 0.01 in all cases (Figure 2).

## DISCUSSION

The main finding of this study is that women with COPD-BS compared with COPD-TS with similar FEV1% had higher eosinophilic inflammatory markers and cytokine levels, correlating positively with R5–R20. COPD-BS patients had more IOS indicators of SAD compared with COPD-TS, suggesting the presence of eosinophilic inflammation associated with SAD.^5,10,11,21^ These findings indirectly show the predominance of the Th2 response activation by CD4^+^ Th2 lymphocytes and airway resistance, as described in the pathophysiology of COPD-BS.^10,12^ In two similar studies involving women, higher levels of some inflammatory cytokines reported here were observed in COPD-BS compared with COPD-TS, specifically IL-1ra, IL-4, IL-6, IL-8, periostin, and eotaxin. However, higher IP-10, RANTES, and vascular endothelial growth factor levels were also observed. These studies favour the eosinophilic inflammatory response and airway resistance in COPD-BS compared with COPD-TS.^10,22,23^

Elevated IL-4, IL-13, and IL-17 levels in COPD-BS show Th2 response, which increases blood eosinophil counts and activity and is a potential treatable trait.^24^ His heightened eosinophilic inflammation correlates with a better response to ICS therapy, potentially reducing exacerbations.^9,25^ The increase in IL-13 in COPD-BS is consistent with the results obtained by Golpe et al.,^12^ underscoring its role in allergic inflammation by modulating lymphocyte and myeloid cell responses. Furthermore, IL-13 and IL-17 drive epithelial remodelling and alveolar destruction in COPD pathophysiology. IL-1ra overexpression further boosts IL-13 production, reinforcing bronchoconstriction and bronchial epithelial damage.^26^ These findings could illustrate the complex interplay between cytokines and eosinophils in COPD-BS.

Higher levels of IL-8 have also been reported in BS-related COPD. This cytokine is involved in both pulmonary and systemic inflammation and is linked to TGF-β1 secretion during the active remodelling process in COPD. Furthermore, BS exposure activates pulmonary macrophages and other cells, inducing IL-6, IL-8, MCP-1, MIP-2, and TNF-α production, which contributes to proteolysis and tissue remodelling^27^; still, in sputum, it has been described in lesser activation.^28^ These findings underscore the inflammatory heterogeneity present in COPD-BS, like that observed in COPD-TS.

These results are consistent with mediators’ eosinophilic inflammation predominates in patients with COPD-BS. It has been reported that women with COPD-BS had a more significant bias toward the Th2 response,^10^ higher sputum eosinophil levels,^11^ and higher fractional exhaled nitric oxide levels (39 ppm vs. 27 ppm) than patients with COPD-TS.^12^ However, when systemic eosinophilia was analysed, Olloquequi et al.^9^ did not find more significant peripheral eosinophilia when comparing patients with COPD-BS and COPD-TS. Furthermore, Salvi et al.^17^ found a 16% increase in mean absolute eosinophils in patients with COPD-TS; however, it was not statistically significant.

We have shown that the cytokines implicated in the eosinophils count are positively associated with SAD. SAD in patients with COPD-BS has been evaluated by histological, tomography, and functional methods.^2^ By spirometry, an increase in meso-flows has been shown,^29^ as well as more significant dynamic hyperinflation.^17^ Recently, IOS has been used as a novel non-invasive method to assess SAD.^21^ Our data are consistent with reports by Salvi et al.,^17^ demonstrating that patients with COPD-BS, in comparison with COPD-TS, present greater SAD. The SAD in patients with COPD-BS is due to remodelling and could explain the functional changes characterised by greater bronchial hyperreactivity.^3^

Finally, a precision-based approach could leverage our understanding of differential biology. Based on these findings, we may hypothesise that, in COPD-BS, damaged bronchial epithelium releases *alarm cytokines* (alarmins) that act upstream of the Th2 response and are associated with SAD. By contrast, in tobacco-related COPD, the activation of these pathways may be partly counterbalanced by neutrophil-related cytokines (e.g., IL-1β, IL-6, and IL-23), steering the response toward Th1/Th17 and thus limiting eosinophilic infiltration.^30^ Nevertheless, in patients with COPD secondary to smoking who do develop an eosinophilic endotype, it is reasonable to assume that the same alarmins orchestrate this response, influenced by factors such as infections or environmental pollution. This reflects the dynamic nature of inflammation and the variability in its clinical presentation,^31^ including variability of the Th2 response in exacerbations^32^ and cigarette smoke exposures.^33^ Moreover, studies have reported a higher prevalence of eosinophilic inflammation in COPD-BS (71% vs. 49%),^11^ suggesting a more pronounced Th2-inflammatory pattern in this subtype. Eosinophilia remains one of the treatable traits; recent trials with anti–IL-4/IL-13 therapies have shown improved exacerbation outcomes in tobacco-related COPD. Similar studies in COPD-BS would be necessary to assess any corresponding benefits in this patient subgroup.^34^

Our pilot study had limited numbers of patients and samples, but we found associations in a cross-sectional design. However, limitations include the lack of a detailed evaluation of possible additional causes influencing the number of eosinophils, such as bowel parasitism. The non-inclusion of patients with exposure to BS and TS may limit the generalisation of the results since a good part of the patients with COPD may be maintained with active exposures; in addition, the non-exposure was only self-reported, and there were no biochemical measurements, such as carbon monoxide measurements. Also, excluding patients with active exposure decreased the possibility of reflecting the response of the variability of the expression of alarmins. The history of atopy was only investigated through questioning, which is subject to memory bias. The effect of exposure intensity to BS or TS and the relationship with cytokines could not be measured since this pilot study had limited data.

## CONCLUSION

Our findings suggest that eosinophil counts and cytokines are higher in COPD-BS and positively correlate with small airway resistance measured by IOS. This could indicate some physio-pathological mechanisms related to SAD and a possible treatable feature of COPD-BS. These results require replication and more patients to corroborate these findings.
